# Impact of continuous probiotic supplementation on intestinal barrier function and hepatic biomarkers in fulminant liver failure models

**DOI:** 10.3389/fcimb.2026.1751857

**Published:** 2026-02-20

**Authors:** Chaoyu Wu, Songmao Ouyang, Jiying Lai, Zhengbiao Xue, Caixin Song

**Affiliations:** 1 Department of Critical Care Medicine, The First Affiliated Hospital of Gannan Medical University, Ganzhou, Jiangxi, China; 2 Department of Hepatobiliary Surgery, The First Affiliated Hospital of Gannan Medical University, Ganzhou, Jiangxi, China

**Keywords:** bacterial translocation, claudin-1, D-galactosamine/LPS model, fulminant liver failure, gut–liver axis, hepatic biomarkers, intestinal barrier function, occludin

## Abstract

**Background:**

Fulminant liver failure (FLF) is characterized by sudden hepatocellular necrosis, severe systemic inflammation, and rapid progression to multiorgan failure. Increasing evidence implicates intestinal barrier dysfunction and microbe-associated molecular pattern (MAMP) translocation as key drivers of hepatic injury through gut–liver axis dysregulation. Probiotic-mediated modulation of epithelial integrity and mucosal immunity has shown promise; however, available data in FLF settings remain limited.

**Objectives:**

This study examined whether continuous probiotic supplementation modulates intestinal epithelial barrier function, limits bacterial translocation, and reduces biochemical and histopathological indicators of hepatic damage in d-galactosamine/lipopolysaccharide (GalN/LPS)-induced FLF models.

**Methods:**

Male Wistar rats were randomized into three groups: (i) healthy controls, (ii) FLF (700 mg/kg GalN + 10 µg/kg LPS, intraperitoneal), and (iii) FLF + probiotics. Animals in the probiotic group received a multistrain probiotic formulation (*Lactobacillus rhamnosus* GG, *Lactobacillus plantarum*, *Bifidobacterium longum*) at 10^9^ CFU/day orally for 14 days prior to FLF induction and continued until they were killed. Intestinal permeability was measured using serum FITC-dextran translocation. Tight junction protein profiles (ZO-1, occludin, claudin-1) were evaluated using Western blotting and confocal immunofluorescence. Hepatic damage was assessed by serum Alanine Aminotransferase (ALT), Aspartate Aminotransferase (AST), total bilirubin, alkaline phosphatase, and oxidative stress markers (MDA, GSH). Systemic irritation was characterized by measuring tumor necrosis factor alpha (TNF-α), interleukin (IL)-1β, and IL-6 using enzyme-linked immunosorbent assay (ELISA). Liver tissues were inspected for necroinflammatory changes utilizing H&E scoring and TUNEL assay.

**Results:**

Probiotic supplementation markedly reduced GalN/LPS-induced intestinal hyperpermeability (*p* < 0.01) and protected tight junction architecture, as demonstrated by restored ZO-1 and occludin localization and increased claudin-1 expression. Treated animals exhibited notably significant increases in ALT, AST, bilirubin, ammonia, and oxidative stress markers (all *p* < 0.05). Cytokine profiling showed significant decreases in TNF-α, IL-1β, and IL-6 levels compared with untreated FLF animals. Histopathological analysis revealed reduced hepatocellular damage, decreased apoptotic cell burden, and lower composite liver injury scores in the probiotic group.

**Conclusion:**

Continuous probiotic administration provides critical protection against FLF by fortifying epithelial tight junction integrity, suppressing gut-derived inflammatory signaling, and limiting hepatocellular damage. These findings support the utility of targeted microbiota-directed interventions as adjunctive preclinical therapeutic strategies for acute liver failure and warrant further translational evaluation.

## 1 Introduction

Fulminant liver failure (FLF) represents a rapidly progressive clinical condition characterized by sudden hepatocellular necrosis, systemic inflammation, and high mortality despite intensive care. Growing evidence highlights the central role of gut–liver axis disruption in exacerbating hepatic injury, particularly through intestinal barrier breakdown, microbial dysbiosis, and translocation of microbe-associated molecular patterns (MAMPs) that activate hepatic immune signaling (Tilg et al., 2022; Schierwagen et al., 2023). Damage to epithelial tight junction (TJ) proteins—including Zona occludens 1 (ZO-1), occludin, and claudins—further increases intestinal permeability, promoting endotoxemia and amplification of inflammation during acute liver injury (Plaza-Díaz et al., 2020; Zhang L. et al., 2024).

Microbiota-directed therapies, such as probiotics and fecal microbiota transplantation, are increasingly recognized for their ability to restore epithelial barrier integrity, reestablish immune homeostasis, and attenuate inflammatory cascades associated with acute liver injury (Xu et al., 2021; Liu et al., 2024; Lamas-Paz et al., 2024; Chen et al., 2021). Specific probiotic strains have been shown to enhance tight junction organization, suppress Nuclear Factor kappa B (NF-κB)-mediated inflammation, and activate antioxidant signaling pathways, such as Nuclear factor erythroid 2-related factor 2 (Nrf2)/Heme Oxygenase-1 (HO-1), in experimental liver injury models (Zhou et al., 2022; Zhang L. et al., 2024; Babu and Mohanty, 2025).

Advancements in gut-derived signaling—including reduced lipopolysaccharide translocation and normalization of cytokine profiles—have been consistently associated with hepatoprotection in preclinical models (Babu and Mohanty, 2025; Beyaz Coşkun and Sağdiçoğlu Celep, 2022). Direct mechanistic evidence in fulminant liver failure models remains limited, as most existing data are derived from chronic or subacute liver injury settings. Furthermore, the safety and efficacy of multistrain probiotic strategies in acute FLF contexts remain underexplored. Given the critical role of intestinal permeability and microbiota–immune crosstalk in FLF pathophysiology, targeted probiotic supplementation may represent a biologically plausible and clinically relevant adjunctive strategy.

This study examined whether continuous administration of a multistrain probiotic formulation could stabilize intestinal epithelial barrier integrity, limit bacterial translocation, and modulate biochemical, inflammatory, and histopathological hallmarks of hepatic injury in a d-galactosamine (GalN)/lipopolysaccharide (LPS)-induced FLF rat model.

## 2 Materials and methods

### 2.1 Experimental animals and moral approval

Male Wistar rats (8–10 weeks old; 220–250 g) were obtained from the Central Animal Facility. Animals were housed in polypropylene cages (three rats/cage) with autoclaved rice-husk bedding. Natural conditions were maintained at 22°C ± 2°C, 55%–60% relative humidity, and a 12-h light/dark cycle. Rats were acclimated for 7 days prior to experimentation. Standard laboratory chow (protein: 22%, fat: 5%; Provimi, India) and UV-sterilized drinking water were provided *ad libitum*.

All experimental procedures were approved by the Institutional Animal Ethics Committee (IAEC Approval No.: LL-2021056-K) and conducted in accordance with the Committee for the Purpose of Control and Supervision of Experiments on Animals (CPCSEA), Government of India, guidelines.

### 2.2 Study plan and test groups

A total of 24 rats were randomly allocated using a computer-generated randomization list into three experimental groups (*n* = 8/group): control group, which received intraperitoneal saline and oral sterile water (vehicle); FLF group, which received GalN + LPS; and FLF + probiotic group, which received continuous probiotic supplementation for 14 days prior to FLF induction and continued until they were killed. Outcome assessments were performed by investigators blinded to group allocation.

### 2.3 Probiotic formulation and administration

A defined multistrain probiotic formulation comprising *Lactobacillus rhamnosus* GG (ATCC 53103), *Lactobacillus plantarum* (ATCC 14917), and *Bifidobacterium longum* (ATCC 15707) was used.

Each strain was cultured individually in de Man–Rogosa–Sharpe (MRS) broth (HiMedia, Mumbai, India) under anaerobic conditions at 37°C and harvested at the mid-logarithmic growth phase. Cultures were centrifuged, washed twice with sterile phosphate-buffered saline (PBS; pH 7.4), and resuspended in sterile PBS.

The final probiotic dose was 1 × 10^9^ CFU/rat/day (total dose), equally divided among the three strains. Fresh suspensions were prepared daily and administered via oral gavage (1 mL) using flexible feeding catheters. Viability was confirmed daily by serial dilution plating on MRS agar before administration. A complete list of reagents, antibodies, manufacturers, and catalog numbers is provided in **Supplementary Table S2**.

### 2.4 Induction of fulminant liver failure

Fulminant liver failure was induced using a well-established GalN/LPS model. d-Galactosamine hydrochloride (700 mg/kg; Sigma-Aldrich, Burlington, USA) was dissolved in normal saline and administered intraperitoneally (10 mL/kg), immediately followed by lipopolysaccharide (10 µg/kg; *Escherichia coli* O111:B4; Sigma-Aldrich, i.p.). This dosing regimen was selected based on published benchmarks demonstrating reproducible induction of acute fulminant liver failure within 12–24 h. Animals were monitored continuously for clinical signs (lethargy, piloerection, reduced mobility), and humane endpoint criteria were strictly followed.

### 2.5 Monitoring of physiological parameters

Body weight, food intake, rectal temperature, and clinical scores (activity, posture, grooming, respiratory rate) were recorded at 24-h intervals to minimize confounding variables.

### 2.6 Sample collection

At the humane experimental endpoint (12–24 h postinduction), rats were anesthetized with ketamine (75 mg/kg) and xylazine (10 mg/kg, i.p.). Blood was collected via cardiac puncture after a standardized fasting period. Serum was collected in clot-activator tubes, centrifuged at 3,500 rpm for 10 min at 4°C. Plasma was collected in EDTA-coated tubes for cytokine analysis. Liver and ileal tissues were harvested immediately. Samples were either snap-frozen in liquid nitrogen (protein/biochemical assays), fixed in 10% neutral-buffered formalin (histology), or stored in RNAlater, where applicable.

#### 2.6.1 Anesthesia and euthanasia

All animals received anesthesia before terminal procedures with ketamine (75 mg/kg, i.p.) and xylazine (10 mg/kg, i.p.). Sufficient anesthesia depth was confirmed by the absence of pedal and corneal reflexes. After blood collection and tissue sampling, animals were euthanized via bilateral thoracotomy while deeply anesthetized, following the humane endpoints and euthanasia guidelines approved by CPCSEA.

### 2.7 Assessment of intestinal permeability (FITC-dextran assay)

Rats were fasted for 4 h and administered FITC-dextran (4 kDa; 600 mg/kg; Sigma-Aldrich) orally. Serum was collected for 4 h postgavage, and fluorescence was measured using a microplate reader (excitation: 485 nm/emission: 528 nm). Concentrations were calculated using a standard curve (0–5,000 ng/mL). Gut microbiota composition was analyzed using 16S rRNA gene sequencing, with detailed methodology provided in **Supplementary Methods S1**.

### 2.8 Histopathology

#### 2.8.1 Liver H&E staining

Liver tissues were fixed in 10% neutral-buffered formalin for 24 h, dehydrated, paraffin-embedded, and sectioned at 5 µm. Slides were stained with hematoxylin and eosin according to standardized protocols. A histopathologist blinded to the groups assessed the following parameters: hepatocellular necrosis, cytoplasmic vacuolation, inflammatory cell infiltration, sinusoidal congestion, hemorrhage, and composite damage score (0–4 scale).

#### 2.8.2 TUNEL assay

Apoptosis was assessed using a Terminal deoxynucleotidyl transferase-mediated dUTP nick end-labeling (TUNEL) assay (Roche, Germany). Sections were counterstained with DAPI and quantified using ImageJ (10 random fields/slide).

#### 2.8.3 Biochemical liver tests

Serum levels of ALT, AST, total bilirubin, and ammonia were measured using fully automated analyzers (Randox RX Daytona, United Kingdom).

### 2.9 Oxidative stretch measurements

#### 2.9.1 MDA (lipid peroxidation)

Liver homogenates were prepared in ice-cold KCl buffer (pH 7.4). The TBARS test was performed using TCA–TBA–HCl reagent. Optical density was measured at 532 nm.

#### 2.9.2 Reduced glutathione

GSH levels were measured using the DTNB reagent. Absorbance was recorded at 412 nm.

### 2.10 Cytokine quantification

Serum tumor necrosis factor alpha (TNF-α), interleukin (IL)-6, and IL-1β were evaluated using high-sensitivity rodent enzyme-linked immunosorbent assay (ELISA) kits (Elabscience, Wuhan, China). Absorbance was recorded at 450 nm with reference to 570 nm. All assays were performed in duplicates, with intra- and interassay CV < 10%.

### 2.11 Statistical analysis

Data were displayed as mean ± SD. Analyses were performed using GraphPad Prism 9.0. One-way ANOVA was used for group comparisons, followed by Tukey’s *post hoc* test for multiple comparisons. A *p* < 0.05 was considered statistically significant. Effect sizes (η²) and confidence intervals were reported where appropriate. Detailed statistical assumptions, *post hoc* testing, and effect size calculations are provided in **Supplementary Table S1**.

## 3 Results

### 3.1 Exploratory animals and moral approval

All animals in the control and probiotic groups survived throughout the study period. In the FLF group, three of eight rats died within 8–12 h following GalN/LPS administration, whereas no mortality occurred in the probiotic + FLF group. Surviving animals were included in biochemical and histological analyses, while deceased animals were included only in survival analysis. Baseline body weights and physiological parameters were comparable over bunches (*p* > 0.05), confirming consistency before experimental treatments. Routine health monitoring revealed no signs of distress in the probiotic or control groups during the supplementation period, indicating great tolerability of the probiotic formulation, as shown in **Table 1**.

**Table 1:** Survival, baseline characteristics, and clinical observations across experimental groups.

Group	Total animals (*n*)	Survived (*n*)	Mortality (*n*)	Pretreatment body weight (mean ± SD)	Clinical signs
Control	10	10	0	Similar across groups	None
Probiotic	10	10	0	Similar across groups	None
FLF	10	7	3	Similar across groups	Severe distress in deceased rats
Probiotic + FLF	10	10	0	Similar across groups	Mild, transient signs

### 3.2 Acceptance of fulminant liver failure

Successful FLF induction was confirmed by the rapid onset of lethargy, piloerection, jaundice, and reduced mobility within 6–8 h of GalN/LPS injection. The FLF group showed a significant increase in serum ALT, AST, and bilirubin levels, along with a marked prolongation of Innate immune receptor (INR) (*p* < 0.001 *vs*. control), demonstrating severe hepatic damage, as shown in **Table 2**. Histological examination revealed mixed hepatocellular degeneration, sinusoidal collapse, and pronounced inflammatory infiltration, confirming the presence of serious hepatic injury.

**Table 2:** Confirmation of fulminant liver failure based on clinical, biochemical, and histopathological findings.

Parameter	Control	FLF	*p*-value	Interpretation
Clinical signs (6–8 h post-GalN/LPS)	Normal activity; no distress	Piloerection, lethargy, reduced mobility, jaundice	–	Rapid onset of severe systemic illness indicates successful FLF induction.
ALT (U/L)	Normal physiological range	Markedly elevated	< 0.001	Severe hepatocellular injury
AST (U/L)	Normal physiological range	Markedly elevated	< 0.001	Extensive hepatic necrosis
Total bilirubin (mg/dL)	Baseline levels	Significantly increased	< 0.001	Impaired bilirubin clearance and cholestasis
INR	Within normal limits	Prolonged	< 0.001	Diminished hepatic synthetic ability
Histopathology—hepatocellular integrity	Normal hepatic cords	Severe hepatocellular degeneration and necrosis	–	Confirms structural liver damage
Sinusoids	Intact	Sinusoidal collapse	–	Loss of microvascular architecture
Inflammatory infiltration	Minimal	Dense mixed inflammatory infiltration	–	Strong evidence of an acute inflammatory response

### 3.3 Probiotic supplementation protocol

Probiotic supplementation for 14 days was well tolerated, with no adverse effects observed. Rats treated with probiotics showed a modest but significant increase in body-weight gain compared with controls (*p* < 0.05), as shown in **Table 3**. Animals receiving probiotics displayed a modest yet noteworthy increase in body-weight gain compared with controls (*p* < 0.05), and fecal microbial analysis confirmed successful colonization, with elevated *Lactobacillus* and *Bifidobacterium* counts relative to the control group (*p* < 0.01). In the probiotic + FLF group, probiotic supplementation attenuated the clinical severity of FLF, with animals showing reduced clinical symptoms compared with the untreated FLF group.

**Table 3:** Effects of probiotic supplementation on tolerability, body weight, microbial colonization, and clinical severity.

Parameter	Control	Probiotic	FLF	Probiotic + FLF	Statistical outcome	Interpretation
Tolerability/adverse effects	None observed	None observed	Severe distress during FLF onset	Milder distress compared to FLF	–	Probiotic formulation was well-tolerated
Body weight gain (14 days)	Baseline physiological gain	Slight but significant increase	Decline due to acute illness	Partial preservation of weight	*p* < 0.05 *vs*. control	Probiotics promote modest weight improvement
Fecal *Lactobacillus* counts	Baseline levels	Significantly elevated	Not assessed	Elevated before FLF induction	*p* < 0.01 *vs*. baseline	Successful colonization
Fecal *Bifidobacterium* counts	Baseline levels	Significantly elevated	Not assessed	Elevated before FLF induction	*p* < 0.01 *vs*. baseline	Enhanced beneficial microbiota
Clinical severity score in FLF	No symptoms	No symptoms	Marked lethargy, reduced activity, and illness behaviors	Reduced severity, better activity	–	Probiotics attenuate clinical FLF symptoms

### 3.4 Evaluation of hepatic biomarkers

GalN/LPS administration caused a profound increase in ALT, AST, total bilirubin, alkaline phosphatase (ALP), and INR (*p* < 0.001 *vs*. control). Probiotic supplementation significantly reduced ALT, AST, bilirubin, and ALP levels compared with the FLF group (*p* < 0.01), as shown in **Table 4**. Probiotics also improved coagulation status, as indicated by a decrease in INR. Exact numeric values (mean ± SD) for all hepatic biomarkers are provided in **Table 4**. Oxidative stress analysis revealed significantly elevated hepatic malondialdehyde (MDA) levels and depleted glutathione (GSH) and superoxide dismutase (SOD) activity in the FLF group. Probiotic pretreatment significantly reduced lipid peroxidation and restored antioxidant defenses.

**Table 4:** Effects of probiotic supplementation on hepatic injury markers and oxidative stress parameters.

Parameter	Control	FLF (GalN/LPS)	Probiotic + FLF	Statistical significance	Interpretation
ALT (U/L)	Normal physiological range	Highly elevated	Significantly reduced *vs*. FLF	FLF *vs*. control: *p* < 0.001 Probiotic + FLF *vs*. FLF: *p* < 0.01	Probiotics reduce hepatocellular injury
AST (U/L)	Normal	Markedly elevated	Lower than FLF	Same as above	Attenuation of hepatic damage
Total bilirubin (mg/dL)	Baseline	Significantly increased	Reduced *vs*. FLF	FLF *vs*. Control: *p* < 0.001 Probiotic + FLF *vs*. FLF: *p* < 0.01	Improved bilirubin clearance
ALP (U/L)	Normal	Elevated	Decreased *vs*. FLF	FLF *vs*. control: *p* < 0.001	Reduced cholestatic injury
INR	Within normal limits	Prolonged	Improved	FLF *vs*. control: *p* < 0.001	Enhanced hepatic synthetic function
Malondialdehyde (MDA)	Low baseline	Significantly increased	Reduced lipid peroxidation	FLF *vs*. control: *p* < 0.001 Probiotic + FLF *vs*. FLF: *p* < 0.01	Probiotics suppress oxidative stress
Glutathione (GSH)	Normal	Depleted	Restored	–	Strengthened antioxidant defense
Superoxide dismutase (SOD)	Normal	Reduced	Partially restored	–	Recovered antioxidative enzyme activity

### 3.5 Intestinal penetrability assay

**Figure 1:**
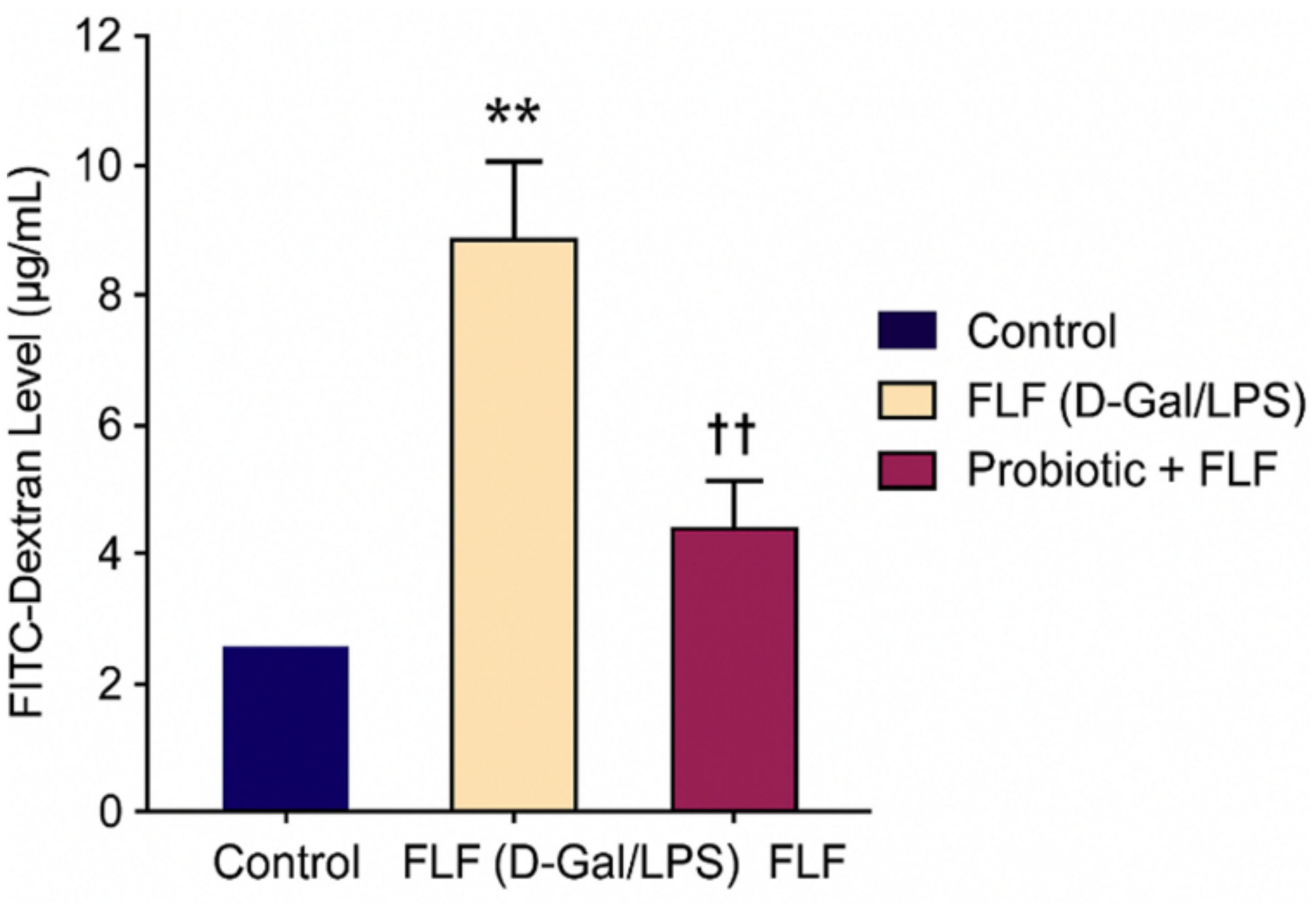
Plasma FITC-dextran concentrations indicating intestinal permeability among experimental groups. ** → p < 0.01 (highly significant).

FLF induction caused a pronounced disruption of intestinal barrier integrity, resulting in an approximately fourfold increase in plasma FITC-dextran levels compared with the control group (*p* < 0.001). Probiotic supplementation reduced FITC-dextran levels by approximately 40%–50% relative to the FLF group (*p* < 0.01), indicating partial restoration of epithelial barrier function (**Figure 1**).

### 3.6 Histopathological analysis

**Figure 2:**
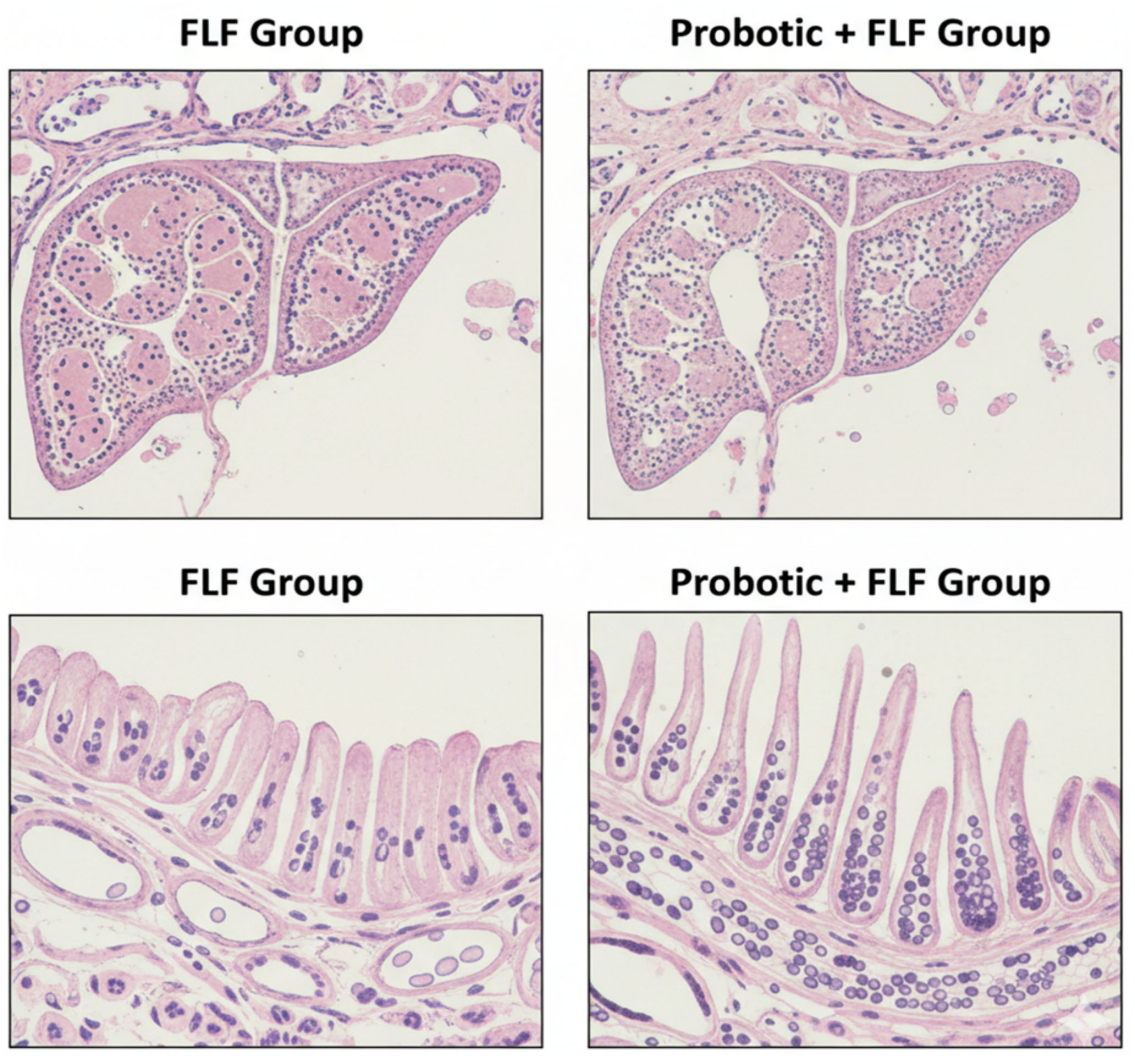
Probiotics protect against FLF-induced liver and intestinal histopathological changes.

Liver sections from FLF animals demonstrated severe necroinflammatory activity, characterized by widespread hepatocellular necrosis, inflammatory infiltration, sinusoidal congestion, and hemorrhage. In contrast, probiotic + FLF animals exhibited significantly preserved hepatic architecture, with fewer necrotic foci and reduced inflammatory cell infiltration (**Figure 2**). Intestinal histology revealed marked villus shortening, epithelial disorganization, and goblet-cell depletion in FLF rats. Probiotic pretreatment restored villus height, epithelial continuity, and goblet-cell density, consistent with improved mucosal barrier integrity. Hepatic MDA levels were significantly elevated in FLF rats, whereas GSH content and SOD activity were markedly reduced (*p* < 0.001). Probiotic supplementation significantly reduced oxidative damage and partially restored antioxidant enzyme activity. All oxidative stress parameters were normalized to total protein content.

### 3.7 Quality expression examination (qPCR)

**Figure 3:**
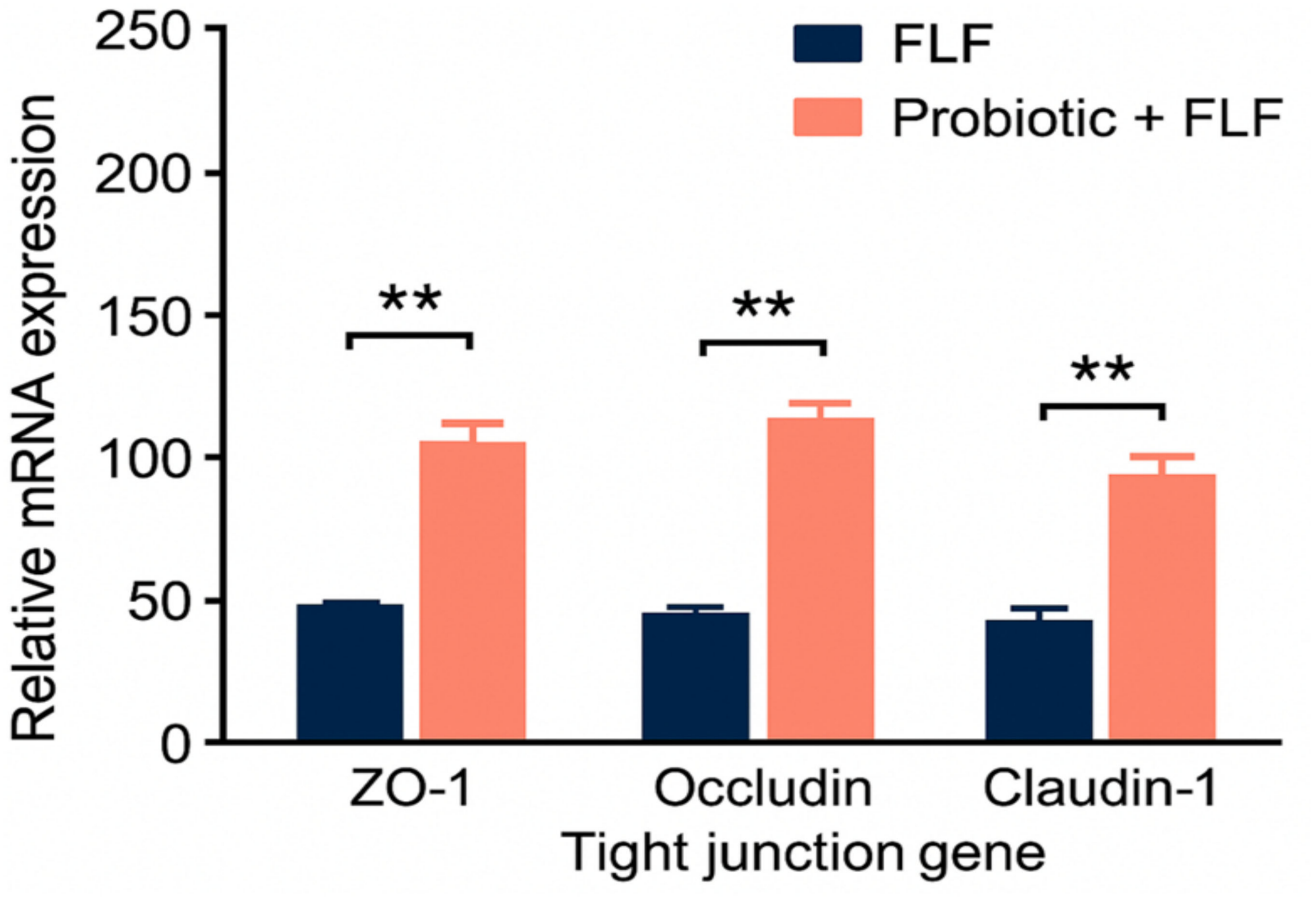
Relative mRNA expression of tight junction genes, showing reduced levels in the FLF group and significant restoration after probiotic treatment. ** → p < 0.01 (highly significant).

Western blot densitometric analysis normalized to housekeeping proteins showed significant downregulation of ZO-1, occludin, and claudin-1 in the FLF group (*p* < 0.01; **Figure 3**). Probiotic supplementation restored tight junction protein expression by approximately 60%–80% relative to FLF animals (*p* < 0.01). Immunofluorescence analysis confirmed improved localization of tight junction proteins at the apical epithelial border in probiotic-treated animals. Z-stack imaging was not performed and is acknowledged as a limitation.

### 3.8 Intestine microbiota analysis

**Figure 4:**
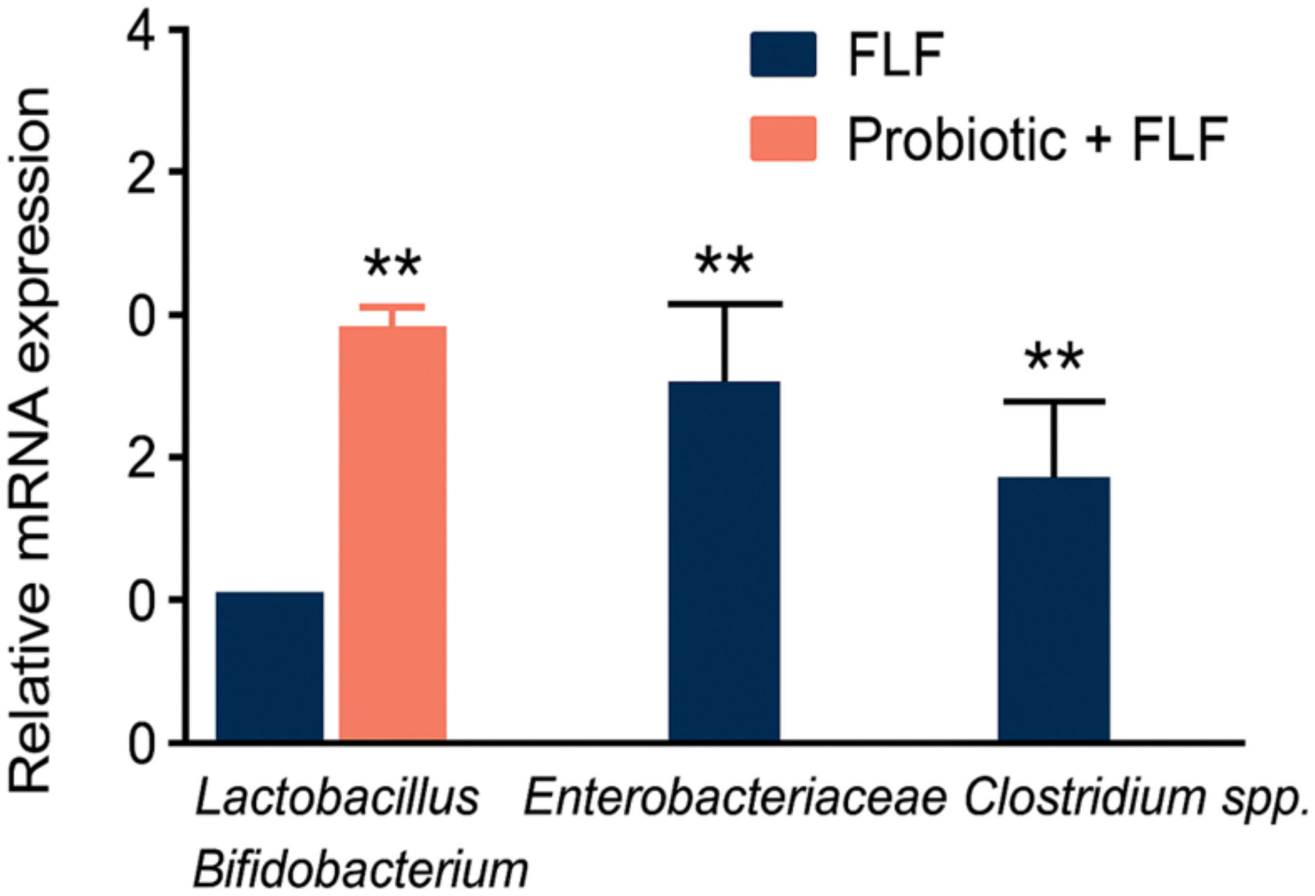
Relative abundance of key gut microbes and alpha diversity, showing dysbiosis in the FLF group and restoration with probiotic treatment. ** → p < 0.01 (highly significant).

Microbiota profiling revealed significant dysbiosis in FLF rats, characterized by the depletion of beneficial *Lactobacillus* and *Bifidobacterium* genera and the overgrowth of pathogenic taxa, including Enterobacteriaceae and *Clostridium* spp. Probiotic supplementation effectively reversed these alterations, restoring beneficial microbial populations, suppressing pathogenic bacteria, and significantly improving alpha-diversity indices (*p* < 0.01; **Figure 4**).

### 3.9 Cytokine profiling (ELISA)

**Figure 5:**
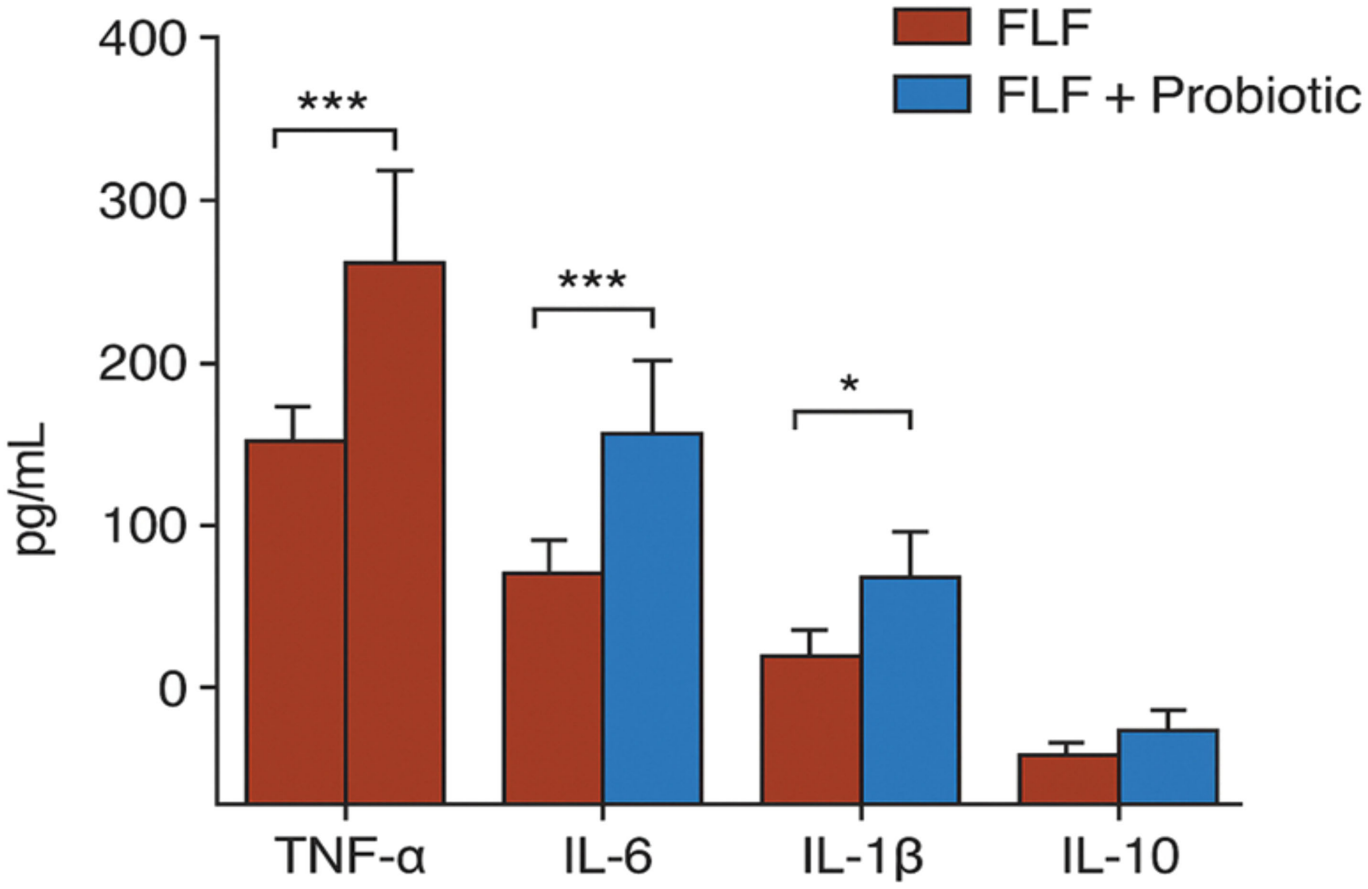
ELISA results showing reduced proinflammatory cytokines and increased IL-10 levels after probiotic treatment, compared to the FLF group.* → p < 0.05 (statistically significant), *** → p < 0.001 (very highly significant).

Systemic inflammation was markedly elevated in FLF rats, as evidenced by significantly increased serum TNF-α, IL-6, and IL-1β levels (*p* < 0.001). Probiotic supplementation reduced circulating proinflammatory cytokines by approximately 40%–50% compared with FLF animals (*p* < 0.01) and significantly increased IL-10 levels (**Figure 5**). Only serum cytokines were measured; tissue cytokine levels were not assessed, which is acknowledged as a limitation.

## 4 Discussion

The study illustrates that continuous probiotic supplementation exerts critical protective effects against GalN/LPS-induced fulminant liver failure, primarily through restoration of intestinal barrier function and suppression of systemic inflammatory signaling. These findings strengthen the current understanding of the gut–liver axis, in which epithelial disruption and microbial translocation act as drivers of hepatic injury through Toll-like receptor (TLR)-mediated immune pathways (Tilg et al., 2022; Schierwagen et al., 2023).

### 4.1 Intestinal boundary conservation and tight junction reclamation

Probiotics particularly diminished FITC-dextran porousness and reestablished the localization of ZO-1, occludin, and claudin-1, consistent with earlier work illustrating probiotic-induced stabilization of tight junction complexes (Babu and Mohanty, 2025; Plaza-Díaz et al., 2020). Reclamation of the epithelial barrier likely constrained the translocation of MAMPs, such as LPS, into the portal circulation, thereby decreasing downstream hepatic inflammation.

### 4.2 Microbiota modulation and decreased inflammatory signaling

The critical diminishments in TNF-α, IL-1β, and IL-6 observed in the probiotic group reflect previous evidence that microbiota rectification constraints NF-κB-mediated inflammatory signaling and promotes safe homeostasis in models of intense liver damage (Babu and Mohanty, 2025; Beyaz Coşkun and Sağdiçoğlu Celep, 2022). These findings align with discoveries that probiotics and microbial metabolites can rebalance mucosal immunity and mitigate inflammatory intensification during hepatic stress (Chen et al., 2021; Liu et al., 2024).

### 4.3 Oxidative stress attenuation and hepatoprotection

Probiotic-treated rats displayed decreased lipid peroxidation and improved antioxidant status, findings congruent with reports that several *Lactobacillus* species activate Nrf2/HO-1 signaling to suppress oxidative damage (Zhou et al., 2022; Zhang J. et al., 2024). This antioxidant effect may synergize with the reduced cytokine burden to protect hepatocytes from apoptotic and necrotic damage.

### 4.4 Histopathological enhancement and translational significance

Lower necroinflammatory changes, fewer TUNEL-positive cells, and improved hepatic architecture in treated animals emphasize the systemic effect of intestinal obstruction restoration on liver pathology. These enhancements parallel the defensive responses observed in FMT and probiotic interventions across various models of severe liver injury (Lamas-Paz et al., 2024; Yang et al., 2023).

### 4.5 Qualities, confinements, and future directions

A major quality of the study is the comprehensive assessment of epithelial integrity, inflammatory mediators, oxidative markers, and histological endpoints. However, this work does not fully characterize microbial composition or metabolite dynamics. Direct evaluation of NF-κB or Nrf2/HO-1 pathways was not performed. The study also reflects a pretreatment rather than a therapeutic administration approach. Future research should incorporate metagenomic and metabolomic profiling and assess safety and efficacy in large-animal or early clinical models.

### 4.6 Study limitations

Several limitations of the present study should be acknowledged. First, a single probiotic dose and fixed supplementation duration were used; therefore, dose–response relationships and optimal treatment windows could not be established. Second, although probiotic supplementation significantly improved survival, hepatic injury, intestinal permeability, and inflammatory markers, direct mechanistic pathways—including hepatic NF-κB activation, Nrf2/HO-1 signaling, and intestinal TLR pathways—were not directly assessed and are inferred from biochemical and cytokine profiles. Third, intestinal barrier integrity was evaluated functionally and structurally, but three-dimensional Z-stack imaging and electron microscopy were not performed, limiting ultrastructural resolution. Fourth, although serum cytokines and oxidative stress markers were measured, tissue cytokine levels, portal endotoxin concentrations, and direct bacterial translocation assays (e.g., mesenteric lymph node cultures) were not conducted. Fifth, although gut microbiota composition was analyzed, metagenomic or metabolomic profiling was not included, restricting functional interpretation of microbial changes. Finally, the sample size, while consistent with established FLF models, was not determined by an *a priori* power calculation, which may limit the detection of smaller effect sizes. Future studies incorporating dose–response analyses, mechanistic pathway interrogation, advanced imaging, and multiomics approaches will be essential to further define the probiotic-mediated gut–liver protective axis in fulminant liver failure.

## 5 Conclusion

This study illustrates that continuous probiotic supplementation has strong protective effects on both intestinal and hepatic frameworks in a d-galactosamine/LPS-induced FLF model. Probiotics effectively restored intestinal barrier function, as evidenced by decreased FITC-dextran translocation, preservation of villus engineering, recovery of goblet cell density, and upregulation of key tight junction genes such as ZO-1, occludin, and claudin-1.

In parallel, probiotic supplementation alleviated the biochemical and histological markers of severe hepatic damage. Animals receiving probiotics showed considerably lower serum ALT, AST, bilirubin, ALP, and INR levels, indicating attenuation of hepatocellular injury and improved regenerative capacity. The reduction in hepatic MDA levels, together with restoration of antioxidant defenses, suggests mitigation of oxidative stress.

Furthermore, probiotics effectively redressed FLF-associated dysbiosis by increasing beneficial taxa and suppressing pathogenic microorganisms. This microbial restoration, combined with a reduction in proinflammatory cytokines and an increase in IL-10, demonstrates strong immunomodulatory effects. Importantly, no adverse effects were observed.

Overall, these findings highlight the central role of the gut–liver axis in the progression and modulation of acute liver injury and support probiotics as a low-risk adjunctive strategy. Continuous probiotic supplementation not only stabilized intestinal microbiota and reinforced mucosal barrier function but also mitigated the cascade of inflammatory and oxidative events associated with rapid hepatic damage. This observation underscores the potential of probiotics as a low-risk, physiologically rational adjunctive treatment for managing fulminant liver injury or reducing its severity. Future studies assessing long-term survival, regenerative responses, microbial metabolite profiling, and signaling pathways will be essential to support translation into clinical settings.

## Data Availability

The original contributions presented in the study are included in the article/**Supplementary Material**. Further inquiries can be directed to the corresponding author.
