# Supplementary material for: Impact of continuous probiotic supplementation on intestinal barrier function and hepatic biomarkers in fulminant liver failure models
Source: Front Cell Infect Microbiol. 2026 Feb 20;16:1751857. doi: 10.3389/fcimb.2026.1751857 (PMC12963265; doi:10.3389/fcimb.2026.1751857)
Supplement: Supplementary file 1 [file Table1.docx]

**SUPPLEMENTARY TABLE S1**

**Statistical Analysis Framework and Effect Size Reporting**

| **Outcome Measure** | **Data Distribution Test** | **Primary Statistical Test** | **Post-hoc Test** | **Significance Threshold** | **Effect Size Reported** | **Software** |
| --- | --- | --- | --- | --- | --- | --- |
| Survival (mortality incidence) | Not applicable | Kaplan–Meier with log-rank test | — | p < 0.05 | Hazard ratio (where applicable) | GraphPad Prism 9.0 |
| ALT, AST, Bilirubin, ALP, INR | Shapiro–Wilk | One-way ANOVA | Tukey’s multiple comparison | p < 0.05 (significant); p < 0.01 (highly significant) | η² (eta-squared) with 95% CI | GraphPad Prism 9.0 |
| FITC-dextran permeability | Shapiro–Wilk | One-way ANOVA | Tukey’s | p < 0.05 | η² | GraphPad Prism 9.0 |
| Cytokines (TNF-α, IL-1β, IL-6, IL-10) | Shapiro–Wilk | One-way ANOVA | Tukey’s | p < 0.05 | η² | GraphPad Prism 9.0 |
| Oxidative stress (MDA, GSH, SOD) | Shapiro–Wilk | One-way ANOVA | Tukey’s | p < 0.05 | η² | GraphPad Prism 9.0 |
| Tight junction proteins (WB densitometry) | Shapiro–Wilk | One-way ANOVA | Tukey’s | p < 0.05 | η² | GraphPad Prism 9.0 |
| Microbiota α-diversity indices | Shapiro–Wilk | One-way ANOVA | Tukey’s | p < 0.05 | η² | GraphPad Prism 9.0 |

**Notes:**

All data are presented as mean ± SD.

Normality was confirmed prior to parametric testing.

Sample size (n = 8/group) was selected based on precedent FLF studies; no a priori power calculation was performed (acknowledged as a limitation).

**SUPPLEMENTARY TABLE S2**

**Reagents, Kits, Antibodies, and Assay Details**

| Reagent / Assay | Manufacturer | Catalog No. | Application |
| --- | --- | --- | --- |
| D-Galactosamine HCl | Sigma-Aldrich (USA) | G0500 | FLF induction |
| Lipopolysaccharide (E. coli O111:B4) | Sigma-Aldrich (USA) | L2630 | FLF induction |
| FITC-Dextran (4 kDa) | Sigma-Aldrich (USA) | FD4 | Intestinal permeability |
| TNF-α ELISA kit (rat) | Elabscience (China) | E-EL-R0019 | Serum cytokine |
| IL-1β ELISA kit (rat) | Elabscience (China) | E-EL-R0012 | Serum cytokine |
| IL-6 ELISA kit (rat) | Elabscience (China) | E-EL-R0015 | Serum cytokine |
| IL-10 ELISA kit (rat) | Elabscience (China) | E-EL-R0016 | Serum cytokine |
| ZO-1 antibody | Invitrogen | 61-7300 | Western blot / IF |
| Occludin antibody | Abcam | ab216327 | Western blot / IF |
| Claudin-1 antibody | Cell Signaling | 13255 | Western blot / IF |
| β-Actin antibody | Cell Signaling | 4970 | Loading control |
| TUNEL assay kit | Roche Diagnostics | 11684817910 | Apoptosis detection |
| MDA assay reagents (TBARS) | HiMedia (India) | MB118 | Lipid peroxidation |
| GSH assay reagents (DTNB) | Sigma-Aldrich | D8130 | Antioxidant status |

Lot numbers are available upon request or can be provided if required by the journal.

**SUPPLEMENTARY METHODS S1**

**Gut Microbiota Composition Analysis**

Fresh fecal pellets were collected from rats at baseline and prior to FLF induction, immediately snap-frozen in liquid nitrogen, and stored at −80 °C until analysis. Total microbial DNA was extracted using a commercial stool DNA extraction kit (Qiagen, Germany) following the manufacturer’s protocol, including bead-beating to ensure efficient lysis of Gram-positive bacteria.

The V3–V4 hypervariable regions of the bacterial 16S rRNA gene were amplified using universal primers and sequenced on an Illumina MiSeq platform (2 × 300 bp paired-end reads). Negative extraction and PCR controls were included to monitor contamination.

Raw sequencing reads were processed using the QIIME2 pipeline. Quality filtering, denoising, and chimera removal were performed using the DADA2 algorithm. Amplicon sequence variants (ASVs) were taxonomically assigned using the SILVA reference database (release 138).

Alpha-diversity metrics (Shannon index, Chao1 richness) and relative taxonomic abundance at phylum and genus levels were calculated. Beta-diversity analyses were performed using Bray–Curtis dissimilarity and visualized by principal coordinate analysis (PCoA). Statistical significance of microbial community differences between groups was assessed using one-way ANOVA for alpha-diversity and PERMANOVA for beta-diversity, with p < 0.05 considered significant.
